# Dissolution behavior of radiocesium-bearing microparticles as a function of solution compositions

**DOI:** 10.1038/s41598-023-31519-6

**Published:** 2023-03-15

**Authors:** Taiga Okumura, Noriko Yamaguchi, Toshihiro Kogure

**Affiliations:** 1 Department of Earth and Planetary Science, Graduate School of Science, The University of Tokyo, 7-3-1 Hongo, Bunkyo-Ku, Tokyo, 113-0033 Japan; 2 Institute for Agro-Environmental Sciences, NARO, 3-1-3 Kannondai, Tsukuba, Ibaraki 305-8604 Japan

**Keywords:** Environmental sciences, Environmental chemistry

## Abstract

More than a decade has passed since the Fukushima nuclear accident in 2011 and contamination around the nuclear power plant is primarily caused by ^137^Cs. One of the materials retaining radiocesium in the environment is radiocesium-bearing silicate glass microparticles (CsMPs), which have not been reported in previous nuclear accidents. Although the prediction of environmental fates of CsMPs is of interest because of their extremely high specific radioactivity, knowledge about their physicochemical properties is still limited. Here we show that the dissolution behavior of CsMPs is comparable to that of silica-rich glass and significantly depends on the surrounding environment. CsMP dissolution experiments were conducted in solutions with various solute components and pH levels at 60 °C. In neutral and basic solutions, the estimated dissolution rate was accelerated by alkali ions such as Na^+^, which is known to play a catalytic role for the dissolution of silica. In contrast, the dissolution in acid was slow even in the presence of alkali ions. The dissolution under acid conditions was possibly retarded by a thin amorphous silica layer formed on the CsMP surfaces. Such characteristics of the dissolution are consistent with that of silica-rich glass. To infer the dissolution behavior of CsMPs in the human body, the dissolution rate in Ringer’s solution at 37 °C was estimated as 1.00 ± 0.37 μm/year.

## Introduction

The nuclear accident at the Tokyo Electric Power Company’s Fukushima Dai-ichi Nuclear Power Plant in March 2011 caused radioactive contamination around the plant. More than a decade after the accident, the main cause of the high air dose rate is attributed to ^137^Cs, which has a relatively long half-life (30.2 years). A large amount of radiocesium (RCs) was originally released in a gaseous form and sorbed to the surfaces of specific minerals such as partially vermiculitized biotite^1,2^. The RCs was strongly fixed to these minerals and thus remains in the soil surface over time^3^. Apart from this form, RCs-bearing silicate glass microparticles (hereafter abbreviated to CsMPs) were first reported by Adachi et al*.* in 2013^4^ and subsequently identified in various contaminated environmental samples, such as soil^5–7^, agricultural materials^8^, plants^9^, and river and ocean sediments^10,11^. Since CsMPs contain uranium and its fission products, they were formed inside the damaged reactor and released into the environment^12^. CsMPs are distinguished from RCs-sorbed minerals by their very high specific radioactivity (10^8–9^ Bq/mm^3^) and small size (a few micrometers). CsMPs smaller than 0.5 μm in diameter were also found^13^. CsMPs must have been widely spread as they were discovered in areas more than 250 km from the nuclear plant^14,15^. The present study focuses on CsMPs with diameters of a few micrometers, but larger silicate glass particles (40–400 μm) containing RCs have also been found in the vicinity of the nuclear plant. The differences of the properties and origins between these two types of the radioactive particles are discussed in a review paper^16^.

Knowledge about physicochemical properties of CsMPs is still limited^17–19^. One of the properties of our interest is their dissolution behavior in the environments. As mentioned above, CsMPs mainly consist of silicate glass containing Na, Cl, K, Fe, Zn, Rb, Sn, and Cs^8,20^. The dissolution behavior of silicate glass is an orthodox topic and a number of results have been reported to date. However, since the composition of CsMPs is specific and there are no analogous glasses, it is probably a shortcut and certain way to investigate their dissolution properties using CsMPs themselves. Although the particles are so tiny, their dissolution rate can be approximately monitored by the loss of their radioactivity. Our previous dissolution experiments indicate that CsMPs dissolve far more rapidly in seawater than in pure water. At 13 °C (the approximate annual mean temperature in Fukushima City), the dissolution rate is approximately one order of magnitude higher in seawater than in pure water^18^. Further dissolution experiments showed that CsMPs dissolve as slowly in acidic solutions as in pure water^9^. On the contrary, Suetake et al*.*^19^ reported that the dissolution rate of CsMPs in seawater and pure water was almost identical, which is controversial to the property of common silicate glasses that dissolve faster in seawater than in pure water^21,22^. Moreover, comprehensive dissolution behavior of CsMPs should be elucidated, in order to discuss the dynamics of CsMPs in various environments. Since numerous knowledge about the dissolution of other silicate glasses has been obtained to date (e.g., Gin et al.^23^), the obtained dissolution characteristics of CsMPs can be compared to these accumulated knowledge to predict the environmental fates of CsMPs. Considering the minute size of CsMPs, solutions surrounding them in the environment will be far from equilibrium, except those in soils where the interstitial water is saturated with silica. Therefore, the relatively rapid initial dissolution rate proposed in previous studies can be comparable with the dissolution of CsMPs. The initial dissolution rate of silicate glass has been reported to increase in both acidic and basic regions^24^. It is necessary to investigate whether this trend is also true for CsMPs, and whether their dissolution rates are affected by solution compositions. In this study, CsMP dissolution experiments are conducted in solutions with various solutes and pH levels, revealing that the dissolution behavior of CsMPs is comparable with that of silica-rich glass. Furthermore, owing to their sub-micron to micron size, CsMPs can be suspended in the atmosphere and widely transported as an aerosol^14^. These microparticles are easily re-suspended, risking internal exposure by inhalation. In fact, CsMPs were found on masks worn during cleaning activity in residential areas near the nuclear plant^25^. To infer the dissolution behavior of CsMPs in the human body, we conducted another CsMP dissolution experiment in a simulated body fluid at the human body temperature (37 °C).

## Methods

### Dissolution experiments of multiple CsMPs

The dissolution experiments were conducted for “multiple CsMPs” attached to a non-woven fabric cloth laid on a vegetable field in Fukushima Prefecture. After approximately six months following the accident, the cloth was removed from the field, cut into (15 × 15) mm^2^ pieces, and exposed to an imaging plate (IP; BAS-2500, Fujifilm) for 10 min. Multiple CsMPs were confirmed as bright spots in the read-out images. The RCs sorbed to the mineral particles was removed by immersing the cloth fragments in 20 mL of 0.1 M hydrochloric acid at 90 °C for 24 h. After this treatment, most of the RCs-bearing materials attached to the cloth fragments were considered as CsMPs^9^. The fragments were tightly folded to a size of (2 × 2) mm^2^ and their ^137^Cs radioactivity was measured by a germanium semiconductor gamma-ray spectrometer (GCW2523S, Canberra). The ^137^Cs radioactivity was corrected to those on 14 March of 2011. The detection efficiency was calibrated with a filter paper onto which a ^137^Cs solution (CZ005, Japan Radioisotope Association) of 100 Bq was dropped and dried. After the measurements, the cloth fragments were wrapped in a hydrophilic Teflon filter (pore size 0.1 μm, H010A025A, Advantec) to prevent detachment of CsMPs from the cloth. The wrapped cloth fragments were immersed in 20 mL of various solutions (see Table 1) and placed at 60 °C. The temperature of 60 °C was adopted in this study because the temperature was suitable for rapidly determining the dissolution behavior of CsMPs based on the previous research^18^. The solutions were periodically exchanged and the radioactivity of ^137^Cs eluted into the solutions was measured using a germanium detector. The detection efficiency of the solution analysis was calibrated with a ^137^Cs standard solution (CZ005) supplied by the Japan Radioisotope Association.

**Table 1:** Solutions used in the dissolution experiments.

Solution	Preparation method
Citrate buffer	Mixed solution of 0.1 M citric acid solution and 0.1 M trisodium citrate solution with pH adjusted to 3.0
Ringer’s solution	A commercial product (SOLYUGEN F INJECTION, KYOWA CritiCare)
Seawater	A commercial product (Daigo’s Artificial Seawater SP, Nihon Pharmaceutical)
Carbonate buffer	Mixed solution of 0.1 M sodium carbonate solution and 0.1 M sodium bicarbonate solution with pH adjusted to 9.7 or 10.0
Carbonate buffer + NaCl	Mixed solution of 0.1 M sodium carbonate solution, 0.1 M sodium bicarbonate solution, and 5 M sodium chloride solution with pH adjusted to 9.7 or 10.0 (final concentration of sodium chloride solution adjusted to be 0.5 M)
Hydrochloric acid	0.001 M HCl (pH 3.0)
Pure water	Ion-exchanged water (pH 5.2 because of CO_2_ absorption from the atmosphere)
Tris–HCl buffer	Tris–HCl buffer solution with pH adjusted to 8.3

### Dissolution experiments of single CsMP

The structural changes of the CsMPs caused by dissolution were investigated in another experiment using isolated single CsMPs. The individual CsMPs were collected as previously described^26^. The CsMPs attached to the non-woven fabric cloth were transferred to 10 mL of ion-exchanged water via ultrasonication. After removing the cloth from the water, the water was divided into 0.5-mL aliquots. The aliquots containing CsMPs were identified by measuring their radioactivity with an automatic gamma counter (Wizard2480, PerkinElmer). The volume of each selected aliquot was increased to 10.5 mL by adding 10 mL of ion-exchanged water. The aliquots were then subdivided into 20 0.5-mL and rechecked for CsMPs. After several repeats of this process, individual CsMPs were isolated from other unrelated particles. The water suspending the CsMP was dropped onto a plastic plate previously coated with carbon and dried at ambient temperature. The CsMPs were observed using a scanning electron microscope (SEM; S-4500, Hitachi) equipped with an energy-dispersive X-ray spectrometer (EDS; UltraDry and NORAN System 7, Thermo Scientific), and their radioactivity was determined using a germanium detector. The CsMPs on the plastic plates were immersed in 20 mL of various solutions with varying degrees of acidity and placed at 60 °C. The radioactivity of ^137^Cs eluted to the solutions was periodically measured as described above. After the immersion period, the CsMPs on the plates were recovered and dried at ambient temperature, observed using SEM, and subsequently thinned to an electron-transparent thickness using a focused ion beam system (FIB) with a micro-sampling unit (FB-2100, Hitachi). These thin specimens were analyzed using a scanning transmission electron microscope (STEM; JEM-2800, JEOL) operated at 200 kV with a silicon drift detector for EDS analysis (X-Max^N^ 100TLE, Oxford Instruments).

### Dissolution experiments in Ringer’s solution at 37 °C

To simulate the dissolution environment in the human body, we adopted Ringer’s solution as a simulated body fluid. The composition of Ringer’s solution is listed in Supplementary Table S1. Unlike the previous study, we did not use the simulated lung fluid because the solution is supersaturated with phosphates and precipitates are formed during the experiment, which may change the solution properties^19^. We preliminarily treated two fragments of non-woven fabric cloth with hydrochloric acid as described above. The radioactivity changes were monitored by IP autoradiography in this experiment to ensure whether individual CsMPs were dissolved in Ringer's solution. During the measurements, the cloth fragments were contacted with the IP in an enclosure of lead blocks for 72 h to determine the distribution of radioactive particles. The radioactivity of the particles was then estimated from the integrated photostimulated luminescence intensity in the IP read-out image (FLA-7000, Fujifilm) calibrated by the radioactivity of CsMPs, which had been preliminarily determined with a germanium detector. Each fragment was immersed in 20 mL of Ringer’s solution and then placed at 37 °C. After immersion, the fragments were recovered from the solution, washed three times with ion-exchanged water, and dried at ambient temperature. The radioactivity change of each radioactive particle was determined by IP autoradiography of the fragments following the same process.

## Results and discussion

### Dissolution rates of CsMPs in various solutions

The changes of ^137^Cs radioactivity in the CsMPs before, during, and after immersion in various solutions are summarized in Supplementary Tables S2 and S3, and Supplementary Fig. S1 plots the residual ^137^Cs radioactivity as a function of immersion time based on the results shown in these tables. Although the CsMP radioactivity (or the number of particles) was different among the experiments, the decrease trends are approximately identical for each solution. Accordingly, the solutions were always far from equilibrium because the glass-surface-to-solution-volume (*S*/*V*) ratio was less than 10^−6^ m^−1^. In addition, the slopes of the plots are approximately linear in all solutions, indicating that there are no significant changes in the dissolution rates during the immersion periods. Therefore, the pretreatment with hydrochloric acid had little effects on subsequent dissolution experiments. The decrease in radioactivity was converted to a decrease in radius (*r*) of the CsMPs as previously reported^18^. In the dissolution experiments of single CsMPs, the radii of the CsMPs were measured from SEM images of the CsMPs before dissolution; in the experiments with multiple CsMPs, the radii were unknown so were originally assumed as 1.06 μm, the average radius of 10 CsMPs of which we have measured both size by SEM and radioactivity by the germanium detector.

**Figure. 1:**
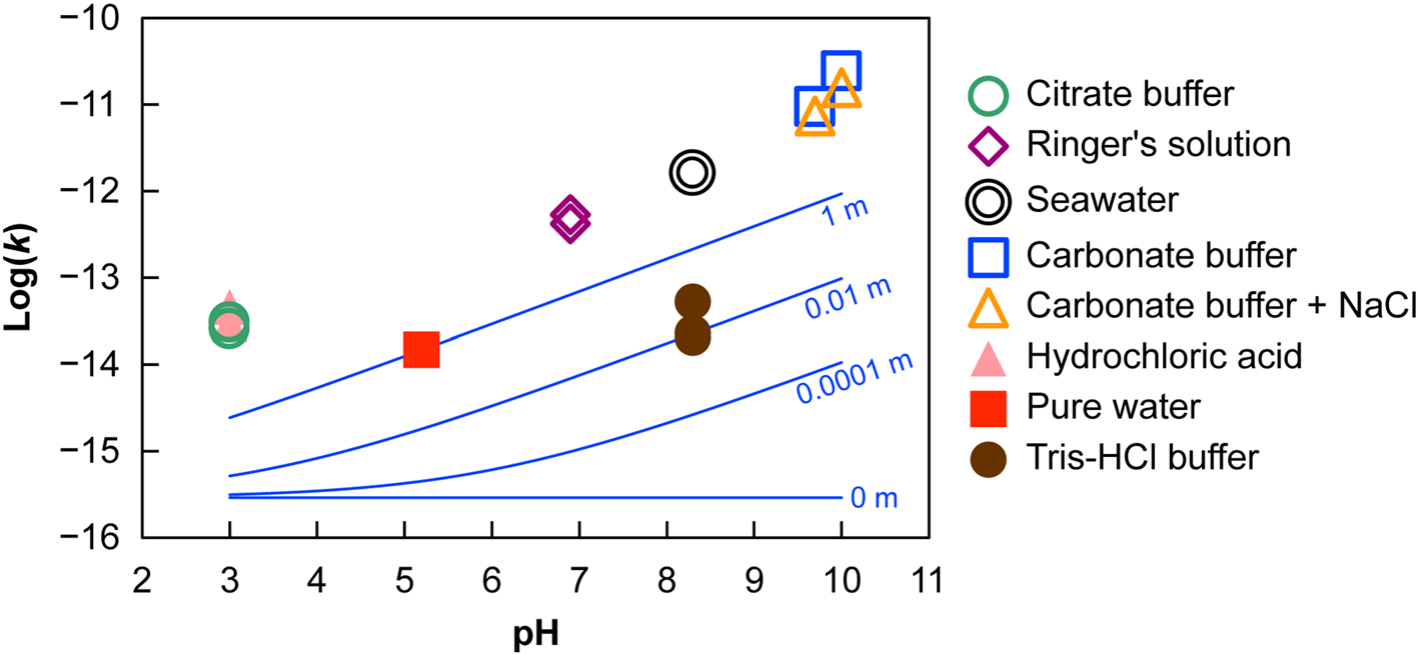
Dependence of dissolution rates (*k*) on pH at 60 °C, where *k* (m/s) is the decrease rate of the CsMP radii in the presence (open symbols) and absence (closed symbols) of sodium ions. Blue lines plot the calculated dissolution rates of silica glass in the solutions containing sodium ions at different molar concentrations (indicated on the plots). The rate equation given in the literature^29^ was used in this calculation.

Supplementary Table S4 summarizes the dissolution rates *k* (m/s) of the radii of the CsMPs and Fig. 1 plots the dependence of *k* on pH in solutions with and without sodium ions. Sodium ion concentrations in each solution are also listed in the table. It should be noted that the dissolution rates contain some uncertainty and may be reduced by half, as evaluated in the previous research^18^, considering the presence of CsMPs where Cs is concentrated near the surface^27,28^. In that case, the logarithm of *k* becomes smaller by approximately 0.3. In the absence of sodium ions, the dissolution rate did not largely depend on pH. The dissolution rate of CsMPs was significantly higher than that of silica glass^29^, probably because CsMPs contain a large amount of alkali elements and most of iron is divalent, which was confirmed using scanning transmission X-ray microscopy in the previous reseach^20,30,31^, and relatively slower than those of soda-lime glasses^32^ (Supplementary Table S5). On the contrary, when sodium ions were present, the dissolution rate increased with increasing pH as previously reported for silica glass and quartz^29,33,34^. This trend suggests that sodium ions catalyze the detachment of Si–O–Si linkages. Figure 1 also plots the calculated dissolution rates of silica glass in the solutions containing sodium ions (blue curves). The following rate equation used in this calculation is given in the literature^29^ (*J*: dissolution flux (mol/m^2^ s), *T*: temperature (K), *m*_Na+_: sodium concentration (molal), *a*_H+_: hydrogen ion activity).

$$J=14.62\left({10}^{-\left(\frac{77400}{2.303R}\right)\left(\frac{1}{T}\right)}\right)+8.95\left({10}^{-\left(\frac{77700}{2.303R}\right)\left(\frac{1}{T}\right)}\right)\left(\frac{{m}_{{\mathrm{Na}}^{+}}^{0.490}}{{a}_{{\mathrm{H}}^{+}}^{0.377}}\right).$$

Molar volume of the silica glass was assumed to be 27.3 cm^3^/mol. Although silica glass generally dissolves more slowly than CsMPs, the pH-dependent increase in the dissolution rate in the presence of sodium ions is consistent with the present results. In conclusion, the dissolution behavior of CsMPs is comparable with that of silica-rich glass.

These results imply that CsMPs dissolve very quickly in high-pH solutions such as seawater but less quickly in forests where the pH levels of stemflow and forest soil are generally low^35,36^. Most of the CsMPs that fell into the ocean immediately after the accident have probably dissolved completely at present, but those deposited on forests are expected to remain. Thus, knowing the surrounding environment is important for understanding the dynamics and fates of CsMPs.

### Dissolution behavior of CsMPs in acidic solutions

As stated above, sodium ions accelerated the dissolution rates of CsMPs in neutral and basic solutions but exerted minimal effect on the dissolution rates in acidic solutions. In general, silicate glass in acid dissolves via ion exchange between protons and alkali elements in the glass before the silicate network is hydrolyzed, thus forming an alkali-depletion layer on the glass surface^31^. To investigate whether the same dissolution processes occur in CsMPs, we observed the CsMPs before and after immersion in acidic solutions using electron microscopy.

**Figure 2:**
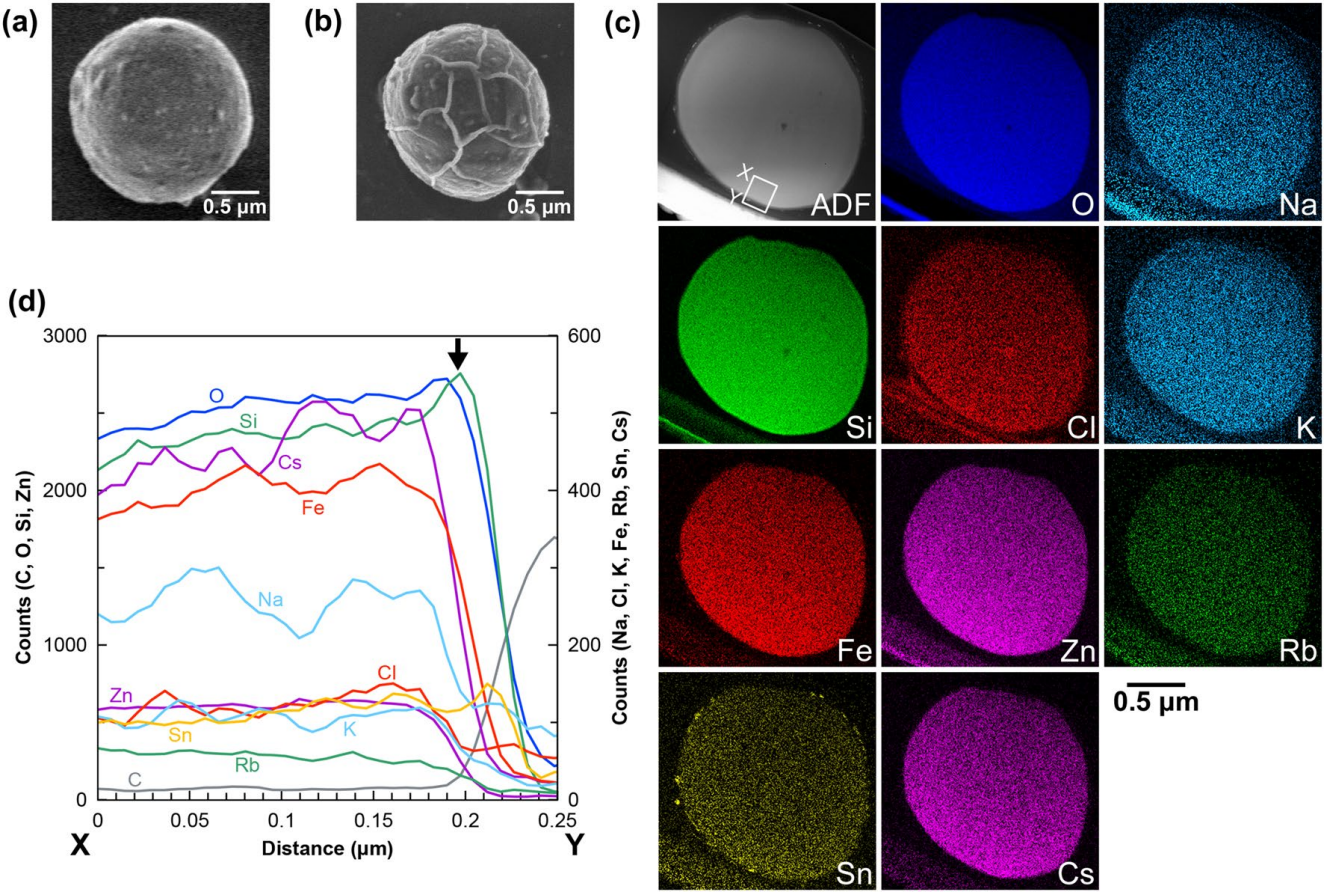
CsMP before and after immersion in hydrochloric acid. SEM (secondary electron) images before (**a**) and after (**b**) immersion. (**c**) Annular dark field (ADF) image and corresponding elemental maps of the CsMP after immersion in hydrochloric acid. (**d**) Profiles of the constituent elements along the line X–Y indicated in the ADF image in (**c**). Arrow points to the profile of an amorphous silica layer formed on the particle surface.

Panels a and b of Fig. 2 are SEM images of a CsMP particle before and after immersion in hydrochloric acid (pH 3.0), respectively. During the dissolution process, the ^137^Cs radioactivity of this particle decreased from 1.52 to 0.78 Bq. After immersion, the particle was slightly decreased in size and many wrinkles appeared on its surface. The post-immersion particle was processed into an electron-transparent thin section using FIB and subjected to STEM–EDS analysis (Fig. 2c). The profiles of the constituent elements along the line X–Y indicated in the ADF image in Fig. 2c are also shown in Fig. 2d. As evidenced by this line profile, a thin layer (a few dozen nanometers thick) composed solely of Si and O was observed on the surface. Transmission electron microscopy (TEM) showed no diffraction contrast in this layer, suggesting that it is amorphous (Supplementary Fig. S2a). In previous research on silicate glass dissolution, such an amorphous silica layer at the interface was suggested to reflect the incongruent dissolution of glass^37^. However, the CsMP dissolution was probably congruent because the concentration of alkali elements did not change between the surface just inside the amorphous silica layer and interior of the CsMP although the incongruent dissolution should cause a concentration gradient. Some researchers proposed that an interfacial layer forms through the congruent dissolution of glass and reprecipitation of amorphous silica^38^. However, in our study, the *S*/*V* ratio was less than 10^−6^ m^−1^ and the solution condition was far from equilibrium, suggesting that reprecipitation was unlikely to occur through dissolution of the CsMPs. The formation mechanism of the amorphous silica layer in CsMPs needs to be revealed in future work.

**Figure 3:**
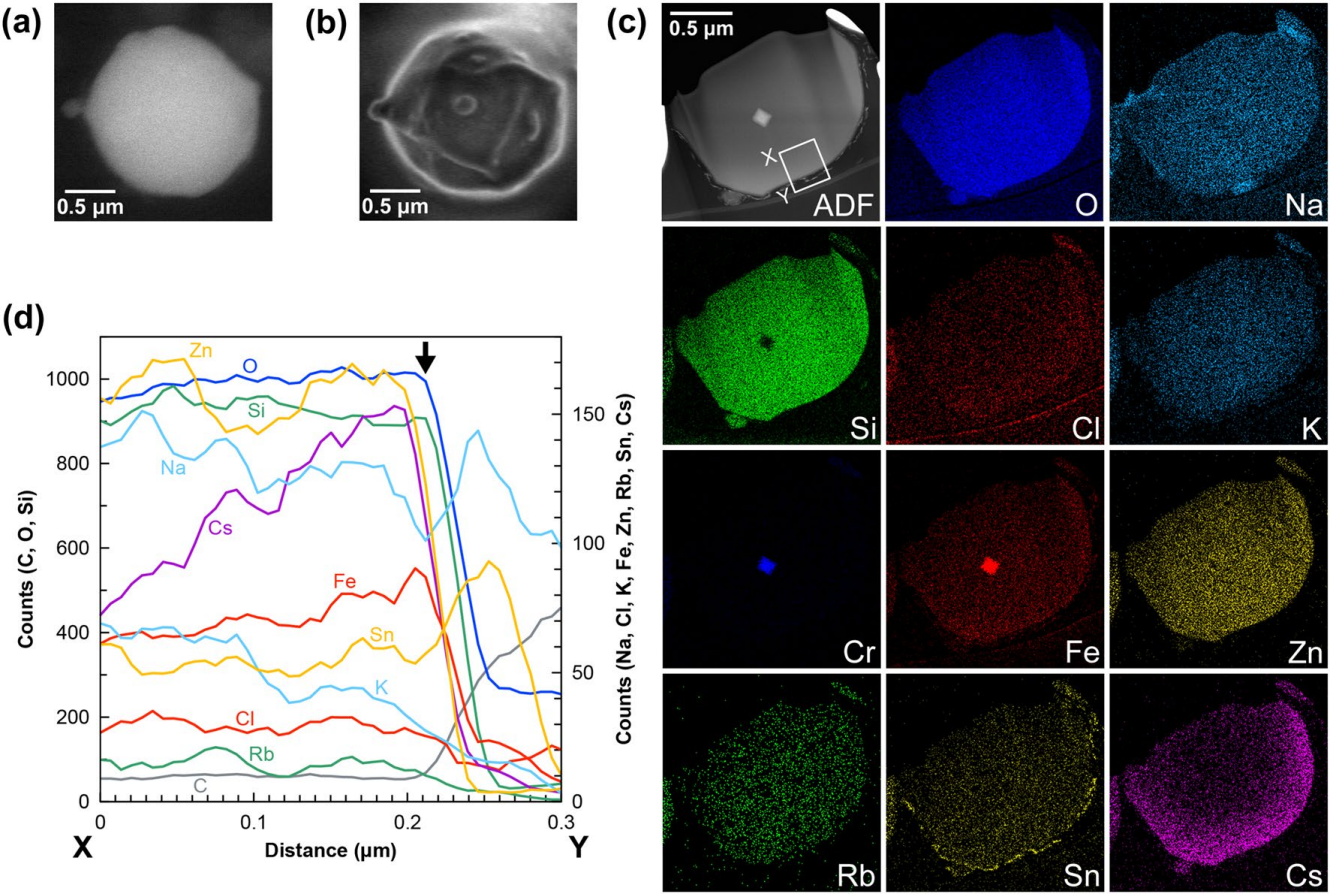
CsMP before and after immersion in citrate buffer solution. SEM images before (**a**) and after (**b**) immersion. Panels (**a**) and (**b**) show back-scattered electron and secondary electron images of the CsMP, respectively. (**c**) ADF image and corresponding elemental maps of the CsMP after immersion in citrate buffer solution. (**d**) Profiles of the constituent elements along the line X–Y indicated in the ADF image in (**c**). Arrow points to the profile of an amorphous silica layer formed on the particle surface.

Figure 3 shows the results of a CsMP immersed in citrate buffer solution (pH 3.0). A back-scattered electron image is shown only for the CsMP before immersion because a secondary electron image could not be obtained due to charge-up. The characteristics of this particle were similar to those of the particle immersed in HCl. After immersion, the ^137^Cs radioactivity of the particle decreased from 0.76 to 0.37 Bq and the surface became wrinkled (Fig. 3a, b). The particle remained almost unchanged in size although its radioactivity halved because the Cs concentration was higher near the surface than in the interior of the particle (see STEM-EDS results in Fig. 3c). Figure 3d shows the profiles of the constituent elements along the line X–Y indicated in the ADF image. A silica layer with a thickness of a few dozen nanometers formed on the surface of the CsMP immersed in citrate buffer solution. This layer was evidenced as amorphous by TEM (Supplementary Fig. S2b). The Na map in Fig. 3c shows Na-rich areas outside of the CsMP, which are probably precipitates formed when the remaining citrate buffer solution dried. Neither increase in Na nor decrease in concentrations of other alkali ions at the CsMP surface were observed even when sodium ions were present in the solution, suggesting that the dissolution proceeded congruently.

For the dissolution experiment of the particle designated as 1906c with Tris–HCl buffer, the results of SEM observations before and after immersion and STEM-EDS analysis after immersion are shown in Supplementary Fig. S3. Like the CsMPs dissolved in pure water^18^, this particle was unchanged except for a decrease in size, and precipitates of iron oxides were observed around the particle. Another particle (MP2020-2) was also immersed in Tris–HCl buffer, but unfortunately it could not be recovered after immersion, thus only the SEM image before immersion is shown in Supplementary Fig. S4. No amorphous silica layer was observed on the CsMPs immersed in seawater, pure water^18^, and Tris–HCl buffer although non-dense precipitates were formed around the CsMPs. Therefore, the dissolution process in acidic solutions must differ from that in neutral and basic solutions. The amorphous silica film might play a role of a passivation layer that affects the dissolution rate in acidic solutions^39^. In addition, tin-rich precipitates on the outer surfaces of both CsMPs were probably formed in the environment before collection^27,40^.

### Dissolution rate of CsMPs in Ringer’s solution at 37 °C

**Figure 4:**
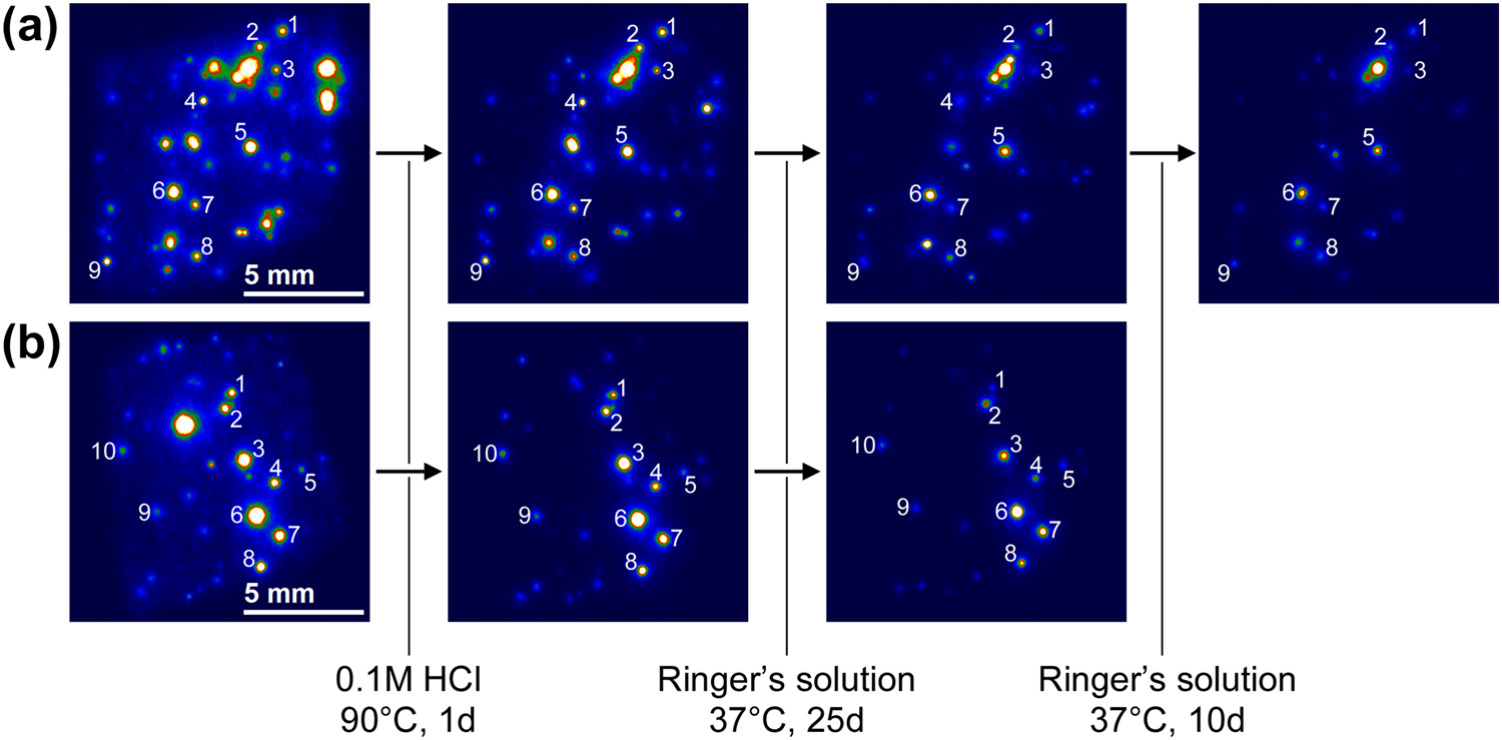
IP read-out images of two fragments of non-woven fabric cloth (**a**,**b**) before and after the immersion treatments (HCl and Ringer’s solution).

Figure 4 shows the IP read-out images acquired before and after each immersion treatment. The uniform luminescence intensity almost disappeared after the acid treatments because the RCs-sorbing minerals lost their radioactivity through dissolution of the mineral surface^9^. Some bright spots completely disappeared after the treatment because the corresponding CsMPs were detached from the cloth. The remaining bright spots were ascribed to CsMPs attached to the cloth fragments. The intensities of all spots were decreased after immersion in Ringer’s solution at 37 °C, confirming the dissolution of CsMPs. The radioactivity of each CsMP after immersion in Ringer’s solution was calculated from the integral intensity of the corresponding spot in the IP read-out images. Radioactivity assessments were made only for isolated spots but not overlapped ones. Some spots disappeared after the treatments because the corresponding CsMPs were completely dissolved or were detached from the cloth during the experiments. The radioactivity changes of the CsMPs after treatment are summarized in Supplementary Table S6. The total radioactivity of the 19 spots shown in this table was originally 1.39 Bq and decreased to 1.17 Bq (residual ratio: 84%) after the pretreatment with hydrochloric acid. Meanwhile, the radioactivity of the CsMP immersed in hydrochloric acid shown in Fig. 2 decreased from 1.52 to 0.78 Bq (residual ratio: 51%), and in this case, an amorphous silica layer of a few dozen nanometers was formed on its surface. Therefore, even though an amorphous silica layer was generated by the pretreatment, it might be less than a few dozen nanometers in thickness and probably have little effect on the subsequent dissolution in Ringer’s solution. As stated above, the decrease in radioactivity was converted into a decrease in radius (*r*) of the CsMPs. The radius *r*, which cannot be determined by IP autoradiography, was calculated from the radioactivity assuming a specific radioactivity of 0.35 Bq/μm^3^, the average value of 10 CsMPs of which we have measured both size by SEM and radioactivity by the germanium detector so far.

The dissolution rate in Ringer’s solution was estimated as 1.00 ± 0.37 μm/year at 37 °C, indicating that CsMPs with radii smaller than 1 μm can completely dissolve in this condition within one year. Thus, the internal dose due to inhalation of CsMPs estimated in a previous study^41^ may be overestimated because it assumed that CsMPs are completely insoluble. In addition, internal dose for Tokyo Electric Power Company employees who worked on site during the accident was periodically monitored with a whole-body counter. The effective half-life of the RCs determined from these measurements was longer than that predicted by the biokinetic model, which was attributed to residual CsMPs inside the body^42^. However, the present study suggests considerable dissolution of the CsMPs within the measurement period. It is worth noting that the intracellular environment of the lungs has a slightly lower pH (~ 4.5)^43^, implying that it will take a longer time for the CsMP dissolution if CsMPs are engulfed by macrophages. Further studies are needed on the dynamics of CsMPs in the human body.

Here we compare the dissolution rates of CsMPs in Ringer’s solution determined in this study and in simulated lung fluid reported by a previous work^19^, in which the ^137^Cs release rates at 25 °C was determined as 4.68, 1.54, and 1.04 × 10^3^ Bq m^−2^ s^−1^ in three experiments with different specimens and conditions. Using the specific radioactivity (Bq/g) and the density of CsMPs (2.6 g/cm^3^) assumed in the previous study^19^, these values were converted to dissolution rates of 0.21, 0.07, and 0.06 μm/year, respectively. These values and the dissolution rates in Ringer’s solution were added to the Arrhenius plot adapted from our previous study^18^ (Supplementary Fig. S5). It is apparent that the dissolution rates are slightly lower in Ringer’s solution than seawater, but those in simulated lung fluid are even lower except for one data point. Although the origin for this discrepancy is not clear, one reason may be different chemical compositions of CsMPs. Silicate glasses are known to have different dissolution rates depending on their chemical compositions^29,44,45^. Another reason may be the different experimental conditions and immersed solutions. Nevertheless, it can be concluded that the dissolution of CsMPs in these solutions proceeds at a certain rate at the human body temperature of 37 °C.

## Conclusions

We conducted dissolution experiments of CsMPs in various solutions and estimated their dissolution rates. Alkali ions such as Na^+^ catalyzed the CsMP dissolution process in neutral and basic solutions but exerted little effect in acidic solutions, probably because dissolution is retarded by the thin amorphous silica layer formed on the CsMP surface under acidic conditions. As silica-rich bulk glasses have similar pH and alkali-ion dependences of their dissolution rates, the dissolution characteristics of CsMPs are considered to be close to them. The dissolution rate of CsMPs in Ringer’s solution at 37 °C was estimated as 1.00 ± 0.37 μm/year, suggesting that CsMPs dissolve at a certain rate in the human body although further research such as in vivo experiments is necessary to clarify the impact on human health. According to these findings, the dissolution behavior of CsMPs in various environments can be predicted from a lot of knowledge about silicate glasses accumulated to date.

## Supplementary Information

Supplementary Information.

## Data Availability

The datasets generated during and/or analyzed during the current study are available from the corresponding author on reasonable request.
