# Supplementary material for: Dissolution behavior of radiocesium-bearing microparticles as a function of solution compositions
Source: Sci Rep. 2023 Mar 15;13:4307. doi: 10.1038/s41598-023-31519-6 (PMC10017807; doi:10.1038/s41598-023-31519-6)
Supplement: Supplementary file 1 — Supplementary Information. [file 41598_2023_31519_MOESM1_ESM.pdf]

Supplementary information for

Dissolution behavior of radiocesium-bearing microparticles as a function of solution compositions

Taiga Okumura [1,*], Noriko Yamaguchi [2], Toshihiro Kogure [1]

[1] Department of Earth and Planetary Science, Graduate School of Science, The University of Tokyo, 7-3-1 Hongo, Bunkyo-ku, Tokyo 113-0033, Japan

[2] Institute for Agro-Environmental Sciences, NARO, 3-1-3 Kannondai, Tsukuba, Ibaraki 305-0864, Japan

* Corresponding author

E-mail: okumura@eps.s.u-tokyo.ac.jp

Table S1. Composition of Ringer's solution (from the manufacturer's data).

	Concentration (mM)
NaCl	103
KCl	4
$CaCl_2 \cdot 2H_2O$	1
$CH_3COONa \cdot 3H_2O$	28
pH adjuster	

Table S2. Summary of dissolution experiments on multiple CsMPs at 60 °C.

Solution	pH	Sample name	Time (h)	^{137}Cs (Bq)	r (μm) [a]
Citrate buffer	3.0	CitB	0	0.41	1.06
			407	0.36	1.01
			887	0.29	0.94
			1607	0.24	0.88
Ringer's solution	6.9	IP-ID25	0	2.90	1.06
			120	1.04	0.75
			240	0.61	0.63
			335	0.38	0.54
	6.9	IP-ID29	0	0.57	1.06
			72	0.37	0.91
			144	0.22	0.77
			216	0.13	0.64
Seawater	8.3	IP-ID23	0	1.06	1.06
			22	0.63	0.89
			44	0.35	0.73
			66	0.22	0.63
			92	0.13	0.52
Carbonate buffer	9.7	CarB	0	0.57	1.06
			4	0.44	0.97
			8	0.26	0.81
			12	0.15	0.67
			18	0.05	0.46
	10.0	CarB10-2	0	0.39	1.06
			6	0.14	0.75
			12	0.00	0.00
Carbonate buffer + NaCl	9.7	CarNa	0	0.48	1.06
			4	0.41	1.00
			8	0.28	0.88
			12	0.18	0.77
			18	0.08	0.59
	10.0	CarNa10-2	0	0.44	1.06
			6	0.21	0.82
			12	0.08	0.60
			18	0.00	0.00
Hydrochloric acid	3.0	HCl-60	0	0.46	1.06
			407	0.39	1.00
			887	0.32	0.94
			1607	0.19	0.79
Pure water	5.2	IP-ID26	0	1.82	1.06
			720	1.55	1.00
			1440	1.33	0.95
			2160	1.14	0.90
			3984	0.93	0.84
Tris-HCl buffer	8.3	Tris-HCl60	0	0.58	1.06
			407	0.44	0.96
			887	0.33	0.87
			1607	0.21	0.75

[a] r: radii of CsMPs

Table S3. Summary of dissolution experiments on single CsMPs at 60 °C.

Solution	pH	Sample name	Time (h)	^{137}Cs (Bq)	r (μm) [a]
Citrate buffer	3.0	1906a	0	0.76	0.85
			576	0.54	0.76
			1200	0.45	0.71
			1920	0.37	0.67
Hydrochloric acid	3.0	a-6	0	1.52	0.96
			360	1.41	0.93
			840	1.25	0.90
			1320	1.06	0.85
			1704	0.78	0.77
Tris-HCl buffer	8.3	1906c	0	0.34	0.62
			750	0.28	0.58
			1200	0.23	0.54
			1650	0.18	0.50
	8.3	MP2020-2	0	3.58	1.35
			480	3.40	1.32
			1152	3.03	1.27
			1848	2.51	1.20

[a] r: radii of CsMPs

Table S4. Dissolution rates (*k*) of the CsMPs at 60 °C.

Solution	Sample name	pH	Na^+ (M)	Log(*k*) (m/s)
Citrate buffer	CitB	3.0	0.054	−13.52
	1906a	3.0	0.054	−13.59
Ringer's solution	IP-ID25	6.9	0.130	−12.38
	IP-ID29	6.9	0.130	−12.27
Seawater	IP-ID23	8.3	0.407	−11.79
Carbonate buffer	CarB	9.7	0.035	−11.03
	CarB10-2	10.0	0.039	−10.61
Carbonate buffer + NaCl	CarNa	9.7	0.535	−11.13
	CarNa10-2	10.0	0.539	−10.80
Hydrochloric acid	HCl-60	3.0	0	−13.34
	a-6	3.0	0	−13.53
Pure water	IP-ID26	5.2	0	−13.83
Tris-HCl buffer	Tris-HCl60	8.3	0	−13.28
	1906c	8.3	0	−13.69
	MP2020-2	8.3	0	−13.65

Table S5. Comparison of dissolution rates of CsMPs and other silicate glasses reported in the literature (solution pH = 7.0 (5.2 for CsMPs); temperature = 60 °C).

Glass	Composition (at%, except O)												Log(*k*) (m/s)
	Na	Mg	Al	Si	Cl	K	Ca	Fe	Zn	Rb	Sn	Cs	
Silica glass				100									−15.54 [a]
Soda-lime glass [b]	36.9			58.5			4.6						−11.27
Soda-lime glass [b]	24.5	2.2	0.2	66.1			6.9						−11.93
Soda-lime glass [b]	22.9	2.6	5.3	63.7			5.5						−13.72
CsMPs [c]	9.2			65.8	2.2	2.8		5.6	7.3	1.4	2.2	3.5	−13.83 [d]

[a] Calculated from a previously derived equation[1] assuming 27.3 cm^3/mol as the molar volume of silica glass. [b] From Perera et al. (1991)[2]. [c] Average composition of two CsMPs (CsMP-Fc and CsMP-HD) reported in Okumura et al. (2020)[3]. [d] From a dissolution experiment in pure water (pH = 5.2).

Table S6. Results of dissolution experiments in Ringer's solution at 37 °C.

Spot number	Time (d)	^{137}Cs (mBq)	r (μm) [a]
a-1	0	46	0.31
	25	23	0.25
	35	13	0.21
a-2	0	50	0.32
	25	22	0.25
	35	18	0.23
a-3	0	30	0.27
	25	11	0.20
	35	7	0.17
a-4	0	37	0.29
	25	16	0.22
a-5	0	92	0.40
	25	59	0.34
	35	39	0.30
a-6	0	100	0.41
	25	73	0.37
	35	48	0.32
a-7	0	36	0.29
	25	14	0.21
	35	10	0.19
a-8	0	36	0.29
	25	21	0.24
	35	16	0.22
a-9	0	36	0.29
	25	11	0.20
	35	7	0.17
b-1	0	38	0.30
	25	9	0.18
b-2	0	51	0.33
	25	29	0.27
b-3	0	134	0.45
	25	49	0.32
b-4	0	47	0.32
	25	22	0.25
b-5	0	13	0.21
	25	8	0.18
b-6	0	262	0.56
	25	153	0.47
b-7	0	73	0.37
	25	57	0.34
b-8	0	57	0.34
	25	32	0.28
b-9	0	15	0.22
	25	8	0.17
b-10	0	21	0.24
	25	11	0.20

[a] r: radii of CsMPs

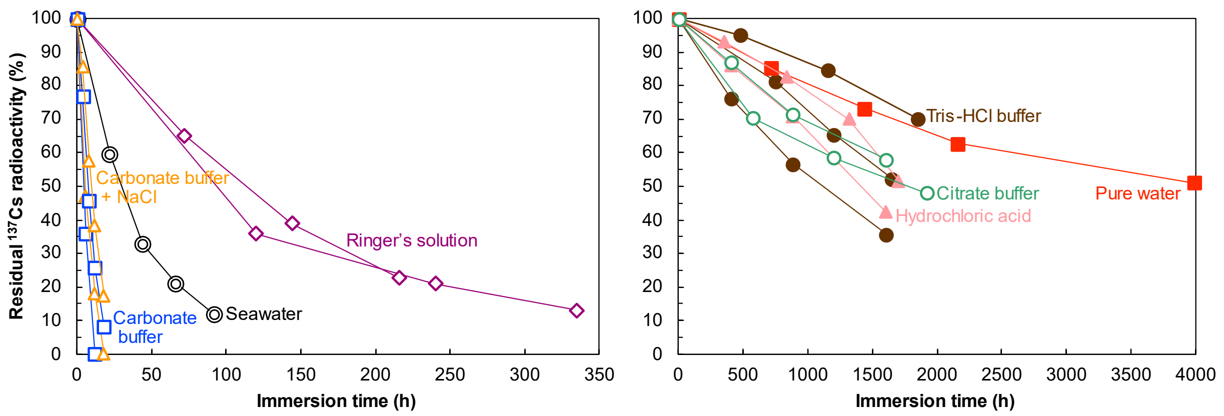

Figure S1. Residual ^{137}Cs radioactivity versus immersion time, plotted based on the results shown in Tables S2 and S3.

(a) (b)

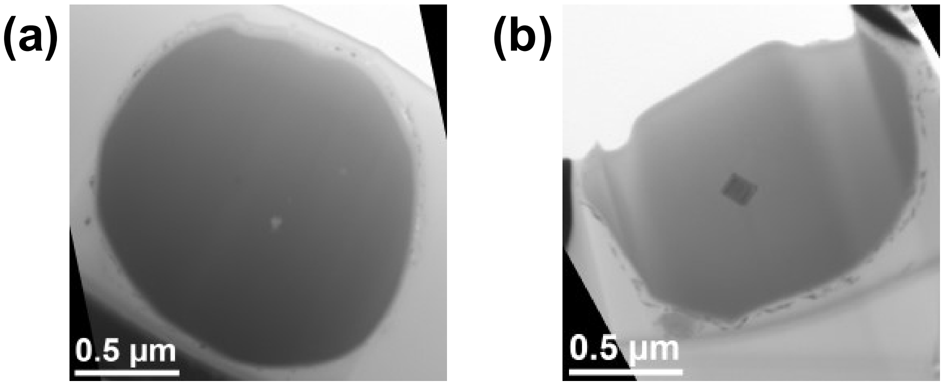

Figure S2. Bright-field transmission electron microscopy images of the CsMPs dissolved in hydrochloric acid (a) and citrate buffer solution (b).

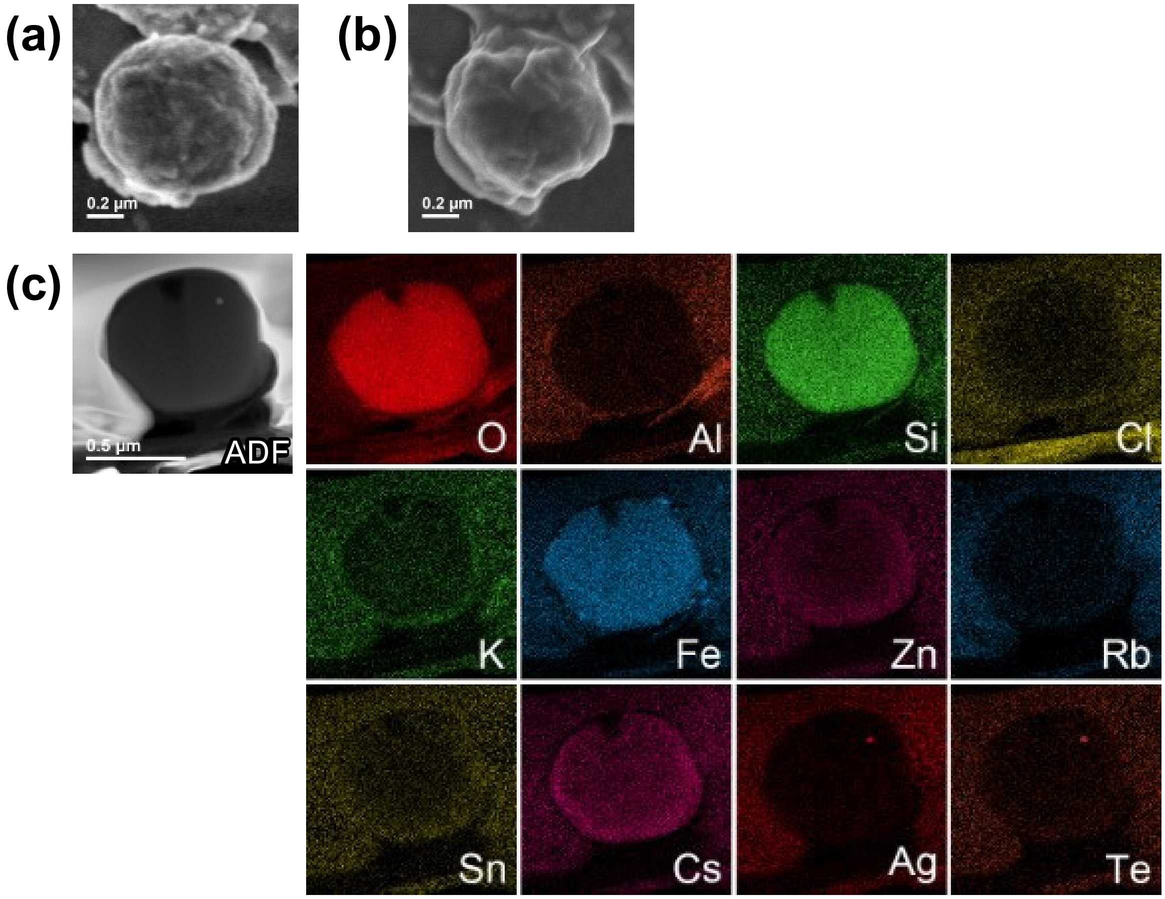

Figure S3. CsMP (1906c) before and after immersion in Tris-HCl buffer solution. SEM (secondary electron) images before (a) and after (b) immersion. (c) ADF image and corresponding elemental maps of the CsMP after immersion. This particle is surrounded by a tungsten protective layer formed during the FIB processing.

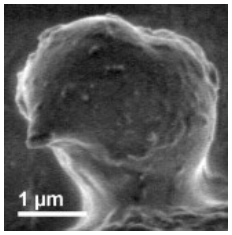

Figure S4. SEM (secondary electron) image of CsMP (MP2020-2) before immersion in Tris-HCl buffer solution.

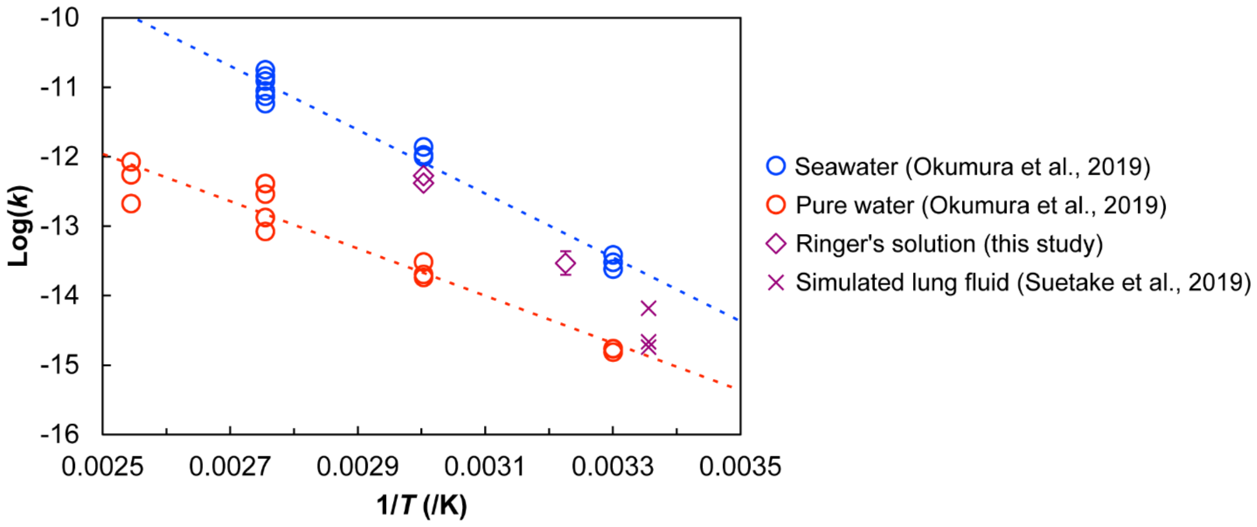

Figure S5. Arrhenius plot of the logarithm of k versus the reciprocal temperature $1/T$. Data for seawater and pure water were adapted from Okumura *et al.* (2019).[4] The dissolution rates in simulated lung fluid were calculated based on Suetake *et al.* (2019)[5].

References

1. Rimstidt, J. D., Zhang, Y. & Zhu, C. Rate equations for sodium catalyzed amorphous silica dissolution. *Geochim. Cosmochim. Acta* **195**, 120–125 (2016).

2. Perera, G., Doremus, R. H. & Lanford, W. Dissolution rates of silicate glasses in water at pH 7. *J. Am. Ceram. Soc.* **74**, 1269–1274 (1991).

3. Okumura, T. *et al.* Reactor environment during the Fukushima nuclear accident inferred from radiocaesium-bearing microparticles. *Sci. Rep.* **10**, 1352 (2020).

4. Okumura, T., Yamaguchi, N., Dohi, T., Iijima, K. & Kogure, T. Dissolution behaviour of radiocaesium-bearing microparticles released from the Fukushima nuclear plant. *Sci. Rep.* **9**, 3520 (2019).

5. Suetake, M. *et al.* Dissolution of radioactive, cesium-rich microparticles released from the Fukushima Daiichi Nuclear Power Plant in simulated lung fluid, pure-water, and seawater. *Chemosphere* **233**, 633–644 (2019).
